# Environmental and Lifestyle Factors in the Risk and Prevention of Rheumatoid Arthritis: A Narrative Review

**DOI:** 10.7759/cureus.93164

**Published:** 2025-09-25

**Authors:** Hubert Ziembicki, Jan Górski, Witold Gajewski, Natalia Skrzypska, Weronika Skoczek

**Affiliations:** 1 Hospital Medicine, University Clinical Hospital, Poznań, POL; 2 Hospital Medicine, Franciszek Raszeja Municipal Hospital, Poznań, POL

**Keywords:** autoimmune diseases, environmental exposures, lifestyle, modifiable risk factors, primary prevention, rheumatoid arthritis

## Abstract

Rheumatoid arthritis (RA) is a chronic autoimmune disease with a multifactorial etiology, driven by a combination of genetic susceptibility and modifiable environmental and exposure factors. While environmental factors such as cigarette smoke, occupational exposures, air pollution, and infections increase the risk of developing RA, lifestyle and dietary factors, including adherence to the Mediterranean diet and adequate vitamin D intake, may offer protective benefits. Systemic factors like obesity, chronic stress, and vitamin D deficiency also contribute to disease onset, highlighting the complex interplay of risk and protective elements in RA development.

This narrative review synthesizes the existing literature on environmental and lifestyle factors associated with RA, focusing on modifiable risks and preventive strategies. A comprehensive literature search was conducted using PubMed and Google Scholar up to 2025, with Boolean combinations of terms related to RA and its environmental and lifestyle triggers. The review highlights the importance of minimizing exposures to environmental triggers and adopting lifestyle modifications, such as a Mediterranean diet, for RA prevention. Although medications like statins and hormone replacement therapy show mixed results, the need for a multifaceted approach combining environmental control, lifestyle changes, and early intervention is emphasized. Further research is needed to refine preventive strategies and deepen understanding of RA pathogenesis, ultimately improving outcomes for individuals at high risk.

## Introduction and background

Rheumatoid arthritis (RA) is a chronic systemic autoimmune disease characterized by persistent inflammation and pain, primarily in the joints, that can lead to progressive damage and loss of function [1]. While RA predominantly affects the joints, it is also considered a systemic disorder capable of involving other tissues and organs. RA represents a substantial global health burden, with its prevalence varying widely across populations and studies. A systematic review of population-based studies by Almutairi et al. found a mean point prevalence of RA of 0.56% globally [2]. Over the last three decades, the global incidence of RA has been on the rise. Zhang et al. reported that the global RA-related incidence rate increased from 11.66 to 13.48 per 100,000 population between 1990 and 2021 [3]. The etiology of RA is not fully understood, but it is now widely recognized as a multifactorial disease resulting from a complex interplay between genetic and environmental factors.

The disease arises from a dysregulated immune response in which the body's own immune cells mistakenly attack the synovial membrane, the lining of the joints [4,5]. This process is characterized by the presence of autoantibodies such as anti-citrullinated protein antibodies (ACPA) and rheumatoid factor (RF), which are considered key markers associated with disease activity and severity. The immune response is triggered by a sequence of events involving the activation of both the innate and adaptive immune systems. This leads to the recruitment of various immune cells, including macrophages and neutrophils, to the site of inflammation. These cells release a host of proinflammatory cytokines, including tumor necrosis factor (TNF)-alpha, Interleukin (IL)-1, and IL-6, which amplify the inflammatory cascade and promote the destruction of cartilage and bone. The bone destruction is driven by an imbalance between bone-resorbing osteoclasts and bone-forming osteoblasts. This irreversible damage results in joint deformity, pain, and significant physical disability [4,6].

The convergence of genetic predisposition and environmental triggers provides a critical mechanistic explanation for the initiation of RA. Genetic factors play a significant role in susceptibility, with certain gene variants, particularly the HLA-DRB1 "shared epitope" gene, being strongly associated with the disease [7]. The crucial link between environmental factors and RA pathogenesis lies in the ability of environmental triggers to induce a specific biochemical process known as citrullination [8]. This is a post-translational modification in which the amino acid arginine is converted to citrulline by peptidylarginine deiminase (PAD) enzymes. This process is typically triggered in areas of inflammation and tissue damage, especially at mucosal surfaces, where external factors may exacerbate the immune response. Citrullination alters the structure of proteins, transforming self-antigens into neoantigens, which the immune system can recognize as foreign. These modified proteins, such as citrullinated fibrinogen or vimentin, may then be targeted by the immune system, initiating an autoimmune response that contributes to RA pathogenesis. Thus, citrullination plays a pivotal role in the activation of the inflammatory pathways that drive the disease.

A broad range of environmental and lifestyle factors are believed to follow this inflammatory pathway. These include inhaled substances such as chronic exposure to cigarette smoke, occupational exposures like crystalline silica, asbestos, and animal and textile dust, and air pollutants [9,10]. Microbial infections have also been implicated in modulating immune responses, potentially influencing the risk of RA [11]. Beyond environmental factors, systemic factors such as obesity and chronic stress are known to promote a state of systemic inflammation, which may predispose individuals to the development of autoimmune conditions [12,13]. Conversely, certain lifestyle factors may have a protective effect, with evidence suggesting that dietary patterns such as adherence to the Mediterranean diet and adequate intake of nutrients like vitamin D could reduce the risk of RA [14,15]. Furthermore, hormone replacement therapy (HRT) and medications such as statins are being explored for their potential role in modulating RA risk, although their effects remain subjects of ongoing investigation [16,17]. Alcohol consumption has also been shown to have a complex relationship with RA, with some studies indicating an inverse association, particularly in ACPA-positive RA [18].

## Review

Methodology

Search Design

This narrative review aimed to summarize the literature on environmental and lifestyle risk factors for RA, focusing on cigarette smoke, occupational exposures, air pollution, infections, and systemic factors. A comprehensive search was conducted using PubMed and Google Scholar for studies from 2015 to 2025, with older studies included when current data were insufficient. The review examined the impact of modifiable factors on RA risk, emphasizing lifestyle interventions for prevention.

A comprehensive search was conducted using PubMed and Google Scholar for studies from 2015 to 2025, with older landmark studies included when current data were insufficient. The search used Boolean combinations of terms such as ("rheumatoid arthritis" OR "RA") AND ("environmental risk factors" OR "cigarette smoke" OR "occupational exposures" OR "air pollution" OR "dietary factors"). Studies were selected based on the following inclusion criteria: peer-reviewed original research (including cohort, case-control, and cross-sectional studies) and meta-analyses examining environmental and lifestyle factors in RA development. Studies were excluded if they met any of the following criteria: focused primarily on genetic factors or the treatment of established RA rather than prevention or risk; were not published in the English language; were non-peer-reviewed article types such as editorials, commentaries, or conference abstracts; or were conducted exclusively on animal models. Because of the significant variability in study methodologies and populations, even after applying these criteria, a formal meta-analysis was not feasible.

Risk of Bias

This review lacked systematic review protocols, introducing potential selection bias. Inclusion criteria were based on the authors' judgment rather than standardized frameworks, and the reliance on PubMed and Google Scholar may have excluded relevant studies. The heterogeneity in study designs and outcome measures limited direct comparisons, so the review prioritized qualitative synthesis. Although efforts were made to minimize bias, the conclusions should be seen as a basis for further research rather than definitive recommendations.

Potential risk factors

Cigarette Smoke

Chronic exposure to cigarette smoke is a major environmental risk factor for the development of many diseases in the modern world. In the context of autoimmune diseases, cigarette smoke affects both adaptive and innate immune responses, leading to alterations in cellular and humoral immunity. These processes may predispose smokers to autoimmune diseases such as RA [9]. The data below summarize the impact of chronic exposure to cigarette smoke on the risk of developing RA.

Hedström et al. conducted a population-based case-control study of 3,655 patients and reported that individuals who had smoked for more than 20 years had nearly a threefold higher risk of developing ACPA-positive RA and a 60% higher risk of ACPA-negative RA [19]. It was noted that the duration of smoking proved to be a more important risk factor than smoking intensity.

In another study, Liu et al. analyzed data from 230,732 women from the Nurses’ Health Study (NHS) and NHS II for the number of cigarettes smoked per day, pack-years, and the incidence of seropositive and seronegative RA [20]. Among women smoking 25 or more cigarettes per day, the risk of developing seropositive RA was 92% higher, whereas smoking intensity did not affect the development of seronegative RA. Moreover, in women exceeding 40 pack-years, the risk of seropositive RA nearly doubled, while pack-years had no effect on the development of seronegative RA.

Similar observations were recorded among passive smokers. Zhang et al., in a systematic review and meta-analysis, noted a 12% increase in RA prevalence in adults and a 34% increase in children exposed to secondhand smoke [21].

These data indicate that cigarette smoke is an important risk factor for the development of RA, regardless of whether the patient smokes personally or is only passively exposed to cigarette smoke. Therefore, for primary prevention of RA, active smoking and exposure to secondhand smoke should be avoided.

Occupational Exposure

Occupational exposure to volatile inhaled substances poses a significant health hazard to workers across various industries. External airborne agents (EEA), once aspirated, are recognized by immune cells as damage-associated molecular patterns (DAMPs). This initiates the formation of large multiprotein complexes known as inflammasomes, which act as signaling platforms for the immune system and contribute to autoinflammatory responses. It has been shown that in patients exposed to EEA, the risk of developing autoimmune diseases was 1.29 times higher than in the control group [10]. The data below summarize the relationship between exposure to specific volatile substances and the risk of an autoimmune response manifesting as RA.

Morotti et al. published a systematic review and meta-analysis of publications from 1960 through November 2019 that demonstrated a statistically significant association between exposure to crystalline silica and the development of both seropositive and seronegative RA [22]. The odds ratio (OR) for seropositive disease was 1.74 (95% CI 1.35-2.25 (I^2^ = 59%)) and for seronegative disease 1.23 (95% CI 1.06-1.43 (I^2^ = 0%)).

Moreover, population-based case-control data, including 11,285 individuals analyzed by Ilar et al., showed the same association but only in men: the OR for seropositive RA was 1.4 (95% CI 1.2-1.6), and for seronegative RA 1.3 (95% CI 1.0-1.5) [23]. It was emphasized that the risk of developing RA increased with the duration of exposure. In the same study, it was shown that men exposed to asbestos also had a higher risk of RA, by 20% for both seropositive and seronegative forms. In women, crude data initially indicated the same association, but after accounting for overlapping factors such as smoking, alcohol use, and exposure to crystalline silica, the association became less significant.

In another population-based case-control study, Ilar et al. analyzed the effects of five types of dust (wood, animal, textile, paper, and flour) on RA development [24]. Exposure to animal dust was associated with increased risk of RA in both men and women: the OR was 1.2 (95% CI 1.1-1.4) for seropositive RA and 1.3 (95% CI 1.1-1.5) for seronegative RA. Additionally, exposure to textile dust showed a dose-dependent association with seropositive RA only (p for trend = 0.014). The effects of wood, paper, and flour dust were not significant.

These data indicate that occupational exposure to inhaled substances such as crystalline silica, asbestos, animal dust, and textile dust is an important risk factor for the development of RA. This suggests that, for primary prevention of RA, exposure to these factors should be minimized by ensuring an appropriate work environment and personal protective equipment.

Air Pollution

Air pollution is a growing problem worldwide, affecting more than half of the global population [25]. In urban areas of the European Union, 18% to 44% of people were exposed to concentrations of atmospheric aerosols with a diameter greater than 10 µm (PM10) that exceeded the EU’s daily permissible value [26]. Additionally, 96% of people were exposed to ozone concentrations exceeding WHO AQG recommendations [27]. It has been suggested that particulate matter (PM), in combination with ozone, is responsible for increased mortality and morbidity from various conditions [28]. The data below summarize the relationship between air pollution and the development of RA.

In a study combining features of cohort and case-control designs that analyzed the effects of PM10, NO₂, SO₂, O₃, and CO on RA development, individuals exposed to ozone and CO had a higher risk of disease. Exposure levels were divided into quartiles with progressively increasing pollutant concentrations. For ozone, the OR was 1.45 (95% CI 1.08-1.96) in the third quartile and 1.35 (95% CI 1.00-1.83) in the fourth quartile. For CO, a positive association was observed in the third quartile, with an OR of 1.57 (95% CI 1.16-2.12). Exposure to PM10, NO₂, and SO₂ did not constitute a risk factor for RA development [28].

Furthermore, a 2019 systematic review and meta-analysis examining the effects of PM10, NO₂, O₃, and CO on RA development also demonstrated a significant role of O₃ as a risk factor. The relative risk (RR) for O₃ was 1.6 (95% CI 1.15-1.18). Exposure to PM10, NO₂, and CO did not show an increased risk of RA [29].

These data indicate that exposure to elevated O₃ concentrations increases the risk of RA development, whereas the effect of CO remains under debate. This suggests that avoiding city environments or outdoor activity during announced smog alerts should be recommended as primary prevention of RA.

Hormone Replacement Therapy

HRT is a medical treatment that restores hormone levels when the body's natural production significantly decreases. It is most commonly prescribed to alleviate moderate to severe symptoms of menopause in women, such as hot flashes, night sweats, vaginal dryness, and mood swings. The primary hormone used is estrogen, which directly addresses these symptoms. For individuals who still have a uterus, a progestogen is always co-administered to protect the uterine lining from the risks of unopposed estrogen [30]. HRT contains therapeutic doses of exogenous sex hormones. They exert diverse effects on tissues and organs, but their impact on the risk of developing RA remains under discussion [16]. The data below address this topic.

A 2023 prospective cohort study demonstrated that patients who had ever used HRT had a higher risk of developing late-onset RA compared with women who had never used it (HR = 1.16; 1.06-1.26) [16]. In contrast, another 2023 cohort study did not show a significant effect of HRT on the risk of RA compared with controls (HR = 1.12; 95% CI 0.998-1.256) [31]. Exceptions included therapies using tibolone or combined progesterone-estrogen therapy, in which the risk of developing RA increased within the first three years of HRT use. The HR for tibolone was 1.33 (95% CI 1.13-1.57), whereas for combined progesterone-estrogen therapy it was 1.24 (95% CI 1.05-1.46).

These data indicate an association between HRT use and RA development, but this relationship should be investigated more thoroughly. Crucially, these findings must be contextualized within the well-established risks associated with HRT, including an increased likelihood of certain cancers and cardiovascular diseases. Therefore, HRT should be used only after a careful, individualized risk-benefit analysis that considers the patient's complete health profile.

Obesity

Obesity is becoming an increasingly serious problem in developed countries. Contributing factors include easy access to inexpensive, highly processed foods, irregular lifestyles, low levels of health education, and insufficient physical activity [12]. Obesity leads to numerous diseases that not only reduce quality of life but may also shorten lifespan. These include cardiovascular, neurodegenerative, and respiratory diseases, prostate cancer, as well as autoimmune diseases, including RA [32].

A cross-sectional study conducted in Israel from 2000 to 2015 assessed the association between obesity and the development of RA using a database from Clalit Health Services. The study included 11,406 RA patients and 54,701 controls matched for age and sex. The results revealed a higher proportion of obesity among RA patients (33.4%) compared with controls (31.6%), with obesity being significantly associated with RA (OR 1.09). This finding supports the hypothesis that obesity, characterized by increased systemic inflammation, contributes to the development of RA [33].

Another study included 55 patients with positive RF who were stratified by body mass index (BMI) and smoking status. In the group with BMI above 25 and among smokers, the risk of developing RA increased from 28% to 60% over 27 months of follow-up. Notably, high body weight proved to be an independent risk factor irrespective of smoking [34].

Obesity is therefore a key risk factor for RA, and its increasing prevalence may contribute to a rise in new cases. Lifestyle modification, including a healthy diet and regular physical activity, is crucial for RA prevention, especially in individuals who are overweight or obese.

Stress

Chronic or intense stress carries numerous adverse health consequences. One effect is increased secretion of glucocorticoids and catecholamines, which disrupts immune balance. As a result, immune tolerance is weakened and humoral responses are heightened, increasing the risk of autoimmune disease [13].

Patients with autoimmune diseases often report episodes of severe stress before symptom onset. This is supported by a French case-control study that included 76 patients with newly diagnosed RA and 76 patients hospitalized for other non-stress-related conditions. Use of a social readjustment scale in both groups showed that RA patients had scores twice as high as controls (167.0 vs 83.3), indicating more frequent exposure to stressful life events. Moreover, a significant association between prolonged stress and RA occurrence was observed, particularly among women [35].

Similar conclusions came from a cohort study conducted between 2007 and 2018 that analyzed the impact of stress on arthritis risk in individuals predisposed to RA. The study included patients with a positive family history or the presence of ACPA. Stress was assessed with the PSS-14 (Perceived Stress Scale), which consists of 14 questions about one’s feelings and thoughts over the past month, with a maximum possible score of 56; higher scores indicate worse perceived well-being. The mean score was 22.4 in the group that developed arthritis versus 20.2 in the group without arthritis. A one-point increase in PSS score raised the risk of arthritis by 6%, even after accounting for other risk factors [36].

Physiological changes induced by stress play an important role in the pathogenesis of inflammatory diseases. Therefore, assessing and controlling stress levels may be helpful in RA prevention, particularly in higher-risk individuals.

Infections

Exposure to microorganisms has long been considered a potential risk factor for the development of RA, and mechanisms plausibly leading to this outcome have been demonstrated [11]. It is difficult, however, to prove this relationship directly, though a growing number of studies show that groups exposed to specific microbes are more susceptible to RA.

The impact of *Mycoplasma* species on the development of arthritis has been observed in animal studies for decades. Until recently, however, there was insufficient evidence to confirm this association in humans. A breakthrough came with a 2019 cohort study from Taiwan [37]. Using health data from 2000 to 2012, investigators identified 116,053 patients diagnosed with *Mycoplasma pneumoniae* and selected 464,212 infection-free controls. The results showed that the risk ratio for developing RA among those with prior infection was 1.37 compared with the control group. Particularly high risk was seen in individuals under 19 and over 65 years of age, with risk ratios of 3.19 compared with controls. The greatest risk was observed within the first two years after infection, when the ratio rose to 4.14 in these groups.

Similar results were obtained in a study examining the association between *Helicobacter pylori* infection and the occurrence of RA [38]. Between 2000 and 2017, 97,533 patients diagnosed with *H. pylori* were identified and matched 1:1 with controls. The study demonstrated a significant association between *H. pylori* infection and the risk of developing RA, with an adjusted risk ratio of 1.45. As in the previous study, the risk of RA was higher in the first years after infection, particularly among individuals under 30 years of age.

These studies suggest a link between certain infections and an increased risk of RA. Further research is still needed to clarify how specific pathogens influence the development of this disease. In the context of RA prevention, appropriate treatment of infections and monitoring of affected patients, especially those with additional risk factors, are important.

Potential protective factors

Mediterranean Diet

In autoimmune diseases such as RA, diet may act as either a protective factor or a risk factor for disease development. This is due to differences in dietary components, which can exert pro- or anti-inflammatory effects [14]. The data below discuss the impact of the Mediterranean diet on the risk of developing RA. This diet is characterized by a high intake of fruits, vegetables, whole grains, legumes, and nuts, with olive oil as the principal source of fat. It also includes moderate consumption of fish and poultry and a low intake of red meat and processed foods.

In a population-based case-control study analyzing the impact of the Mediterranean diet on RA development, an inverse association was observed between adherence to the diet and disease onset. Johansson et al. reported that adherence to the Mediterranean diet reduced the risk of RA by 21% among patients following dietary recommendations (OR 0.79; 95% CI 0.65-0.96) [39]. This association was seen for seropositive RA (OR 0.69; 95% CI 0.54-0.88) but not for seronegative RA (OR 0.96; 95% CI 0.68-1.34).

Although the available studies are limited, the results suggest that the Mediterranean diet may act as a protective factor against the development of RA. This points to the need to implement it, especially in individuals at high risk. However, more studies are required to fully understand this issue.

Statins

Statins are widely used lipid-lowering drugs that help control cardiovascular risk and slow the progression of atherosclerosis. They also exhibit anti-inflammatory effects in patients with inflammatory autoimmune diseases such as RA [17]. In this context, the data below analyze whether the anti-inflammatory action of statins may significantly affect the risk of developing RA.

A 2021 case-control study showed that patients taking statins at the time of the study and in the past had an elevated risk of developing RA compared with those who had never taken them. The OR was 1.12 (95% CI 1.06-1.18) for current users and 1.21 (95% CI 1.13-1.28) for past users. However, these risks returned to baseline after adjustment for coexisting hyperlipidemia. Ultimately, this study did not demonstrate a significant effect of statins on RA development [40]. A 2020 systematic review and meta-analysis comparing RA risk in statin users versus non-users supports these conclusions but indicates the need for further research [41].

Despite their anti-inflammatory properties, statins do not significantly affect the primary prevention of RA, though further studies may reveal more nuanced findings. Therefore, using statins for the primary prevention of RA is unwarranted. Nonetheless, given the small number and low statistical certainty of available studies, further research is advisable to better understand this issue.

Alcohol

Alcohol has long been an integral part of culture. However, studies show that consuming alcohol in amounts exceeding the recommended limits (approximately 20 g for women and 40 g for men) is a significant risk factor for many conditions, such as liver disease, pancreatitis, dementia, and cancers [42].

Conversely, low doses of alcohol may have a beneficial effect on the immune system and thereby reduce the risk of autoimmune diseases, including RA. This mechanism is linked to modulation of the immune system and to effects on the gut microbiota, which produce anti-inflammatory short-chain fatty acids (SCFA) and polyunsaturated fatty acids (PUFA), thereby limiting inflammation [43].

A meta-analysis conducted by researchers at the University of Oxford found that alcohol consumption is inversely correlated with the occurrence of RA associated with the presence of ACPA [18]. Likewise, a prospective study including 347 patients with positive RF but without active arthritis showed that abstaining from alcohol increased the risk of developing RA. In this context, alcohol was identified as a protective factor [44].

These data suggest that alcohol may have a favorable effect on the risk of developing RA. Nevertheless, despite potential benefits for individuals with autoimmune diseases, it is important to remember that excessive alcohol intake increases the risk of other serious conditions, precluding broad recommendations.

Vitamin D

In addition to its well-known role in calcium-phosphate homeostasis and bone mineralization, vitamin D plays a key role in many other bodily systems, helping to maintain homeostasis. One important action is immunomodulation: it inhibits pro-inflammatory Th1 and Th17 lymphocytes and supports regulatory T cells that suppress excessive immune responses. For this reason, vitamin D deficiency may be associated with autoimmune diseases such as RA [15].

A randomized, double-blind trial conducted in the United States confirmed the beneficial effect of vitamin D supplementation in reducing the risk of autoimmune diseases, including RA. Among nearly 26,000 participants, five years of vitamin D supplementation reduced the risk of these conditions by 22% compared with placebo [45]. By contrast, in the same study, omega-3 supplementation did not significantly reduce the risk of autoimmune diseases.

A 2012 meta-analysis of three cohort studies involving approximately 215,000 patients examined the relationship between vitamin D intake and the risk of developing RA [46]. Individuals with the highest vitamin D intake had a 24.2% lower risk of RA compared with those with the lowest intake. These findings indicate that vitamin D deficiency may substantially increase the risk of RA.

A 2023 systematic review and meta-analysis of pre-diagnostic 25-hydroxyvitamin D (25(OH)D) concentrations and the risk of developing RA included seven studies with 15,604 participants [47]. The results were inconclusive, with a pooled relative risk of 0.96 per 25 nmol/L increment in 25(OH)D, indicating no clear association between vitamin D status and RA risk. These findings highlight the need for further research with larger, more diverse populations to better understand the potential role of vitamin D in RA development.

Ensuring adequate vitamin D supplementation is particularly important for RA prevention. In people with additional risk factors or symptoms of deficiency, measuring blood vitamin D levels is recommended, as this may improve the management of deficiency and help prevent autoimmune diseases, including RA.

Key findings

The development of RA is strongly influenced by modifiable risks, with cigarette smoking, occupational exposures, and obesity identified as the most critical environmental and systemic triggers. Protective strategies, particularly adherence to a Mediterranean diet and maintaining adequate vitamin D levels, can substantially mitigate this risk, highlighting actionable pathways for primary prevention. A brief summary of the discussed factors and their influence on RA development is provided in Table 1.

**Table 1:** Selected Modifiable Factors Influencing Rhemuatoid Arthritis Development ACPA, anti-citrullinated protein antibodies; RA, rheumatoid arthritis; BMI, body mass index.

Factor	Description	References
Potential risk factors		
Cigarette smoke	Increases risk, especially for ACPA-positive RA. Duration of smoking is a more significant factor than intensity.	[9, 19, 20, 21]
Occupational exposures	Inhaling substances like crystalline silica, asbestos, and certain dusts elevates the risk.	[10, 22, 23, 24]
Air pollution	Exposure to high concentrations of ozone is associated with a greater risk of developing RA.	[28, 29]
HRT	Certain types of HRT may increase the risk of developing late-onset RA, though the link is still under investigation.	[16, 31]
Obesity	A high BMI is a significant and independent risk factor for RA onset.	[12, 32, 33, 34]
Chronic stress	Disrupts immune balance through physiological changes, increasing susceptibility to autoimmune diseases like RA.	[13, 35, 36]
Infections	Prior infections with specific pathogens, such as *M. pneumoniae* and *H. pylori*, increase subsequent RA risk.	[11, 37, 38]
Potential protective factors		
Mediterranean diet	Adherence to this diet acts as a protective factor, reducing the risk of developing seropositive RA.	[14, 39]
Statins	Despite anti-inflammatory properties, statins do not show a protective effect, and their role remains debated.	[17, 40, 41]
Alcohol	Low-dose consumption is inversely associated with RA risk, particularly the ACPA-positive form.	[18, 43, 44]
Vitamin D	Adequate intake and supplementation help modulate the immune system, significantly lowering the risk of RA.	[15, 45, 46]

## Conclusions

RA is a multifactorial autoimmune disease driven by genetic predisposition and environmental factors such as cigarette smoke, occupational exposures, air pollution, and infections. Smoking remains the most significant modifiable risk factor for both seropositive and seronegative forms of RA. Environmental factors like occupational dust exposure and elevated ozone levels further increase RA risk, highlighting the importance of minimizing these triggers. Systemic factors such as obesity, chronic stress, and vitamin D deficiency also contribute to disease onset, emphasizing the need for comprehensive preventive strategies.

Lifestyle modifications, including adherence to the Mediterranean diet, offer a protective role against RA, while medications like statins and HRT show inconsistent effects on disease risk. A multifaceted approach to RA prevention is essential, incorporating environmental controls, lifestyle interventions, and risk factor management. Continued research is needed to refine prevention strategies, improve understanding of RA pathogenesis, and develop more targeted interventions for high-risk individuals.
